# Magnolol: A Neolignan from the Magnolia Family for the Prevention and Treatment of Cancer

**DOI:** 10.3390/ijms19082362

**Published:** 2018-08-10

**Authors:** Abhishek Manoj Ranaware, Kishore Banik, Vishwas Deshpande, Ganesan Padmavathi, Nand Kishor Roy, Gautam Sethi, Lu Fan, Alan Prem Kumar, Ajaikumar B. Kunnumakkara

**Affiliations:** 1 Department of Zoology, Yashwantrao Chavan Institute of Science, Satara, Maharashtra 415001, India; abhishekranaware@gmail.com (A.M.R.); vydzoo@gmail.com (V.D.); 2 Cancer Biology Laboratory and DBT-AIST International Laboratory for Advanced Biomedicine (DAILAB), Department of Biosciences and Bioengineering, Indian Institute of Technology Guwahati, Assam 781039, India; kishore.banik@iitg.ac.in (K.B.); padmavathi@iitg.ac.in (G.P.); r.nand@iitg.ernet.in (N.K.R.); 3 Department for Management of Science and Technology Development, Ton Duc Thang University, Ho Chi Minh City 700000, Vietnam; 4 Faculty of Pharmacy, Ton Duc Thang University, Ho Chi Minh City 700000, Vietnam; 5 Department of Pharmacology, Yong Loo Lin School of Medicine, National University of Singapore, Singapore 117600, Singapore; phcfanl@nus.edu.sg; 6 Cancer Science Institute of Singapore, National University of Singapore, Singapore 117599, Singapore; csiapk@nus.edu.sg; 7 Medical Science Cluster, Yong Loo Lin School of Medicine, National University of Singapore, Singapore 117599, Singapore; 8 Curtin Medical School, Faculty of Health Sciences, Curtin University, Perth, WA 6009, Australia

**Keywords:** magnolol, cancer, phytochemicals, molecular targets, chemoresistance

## Abstract

The past few decades have witnessed widespread research to challenge carcinogenesis; however, it remains one of the most important health concerns with the worst prognosis and diagnosis. Increasing lines of evidence clearly show that the rate of cancer incidence will increase in future and will create global havoc, designating it as an epidemic. Conventional chemotherapeutics and treatment with synthetic disciplines are often associated with adverse side effects and development of chemoresistance. Thus, discovering novel economic and patient friendly drugs that are safe and efficacious is warranted. Several natural compounds have proved their potential against this dreadful disease so far. Magnolol is a hydroxylated biphenyl isolated from the root and stem bark of Magnolia tree. Magnolol can efficiently prevent or inhibit the growth of various cancers originating from different organs such as brain, breast, cervical, colon, liver, lung, prostate, skin, etc. Considering these perspectives, the current review primarily focuses on the fascinating role of magnolol against various types of cancers, and the source and chemistry of magnolol and the molecular mechanism underlying the targets of magnolol are discussed. This review proposes magnolol as a suitable candidate that can be appropriately designed and established into a potent anti-cancer drug.

## 1. Introduction

Cancer is one of the most lethal diseases and has become a major health concern globally. According to global cancer statistics and GLOBOCAN 2012 (http://globocan.iarc.fr/Default.aspx, accessed on 8 July 2018), approximately 14.1 million people are diagnosed with cancer every year and it accounts for 8.2 million deaths worldwide [1]. The significant advancements made in the past few decades for unravelling the molecular causes of cancer have led to the development of numerous treatment modalities including surgery, radiation, and chemotherapy, but the disease burden still remains a challenge [2,3,4,5,6,7]. On the other hand, these chemotherapeutic agents are also associated with adverse side effects like vomiting, hyper tension, cardiovascular diseases, renal dysfunction and bone marrow destruction along with the development of chemoresistance, which further obscures the treatment procedures and ultimately leads to cancer progression and recurrence [8,9,10,11,12,13,14,15,16,17,18]. Therefore, finding a remedy with minimal side effects, cost effectiveness, easy accessibility and high efficiency is of paramount importance for the effective treatment and management of this outrageous disease.

Mother Nature is the origin of 70% of the pharmaceuticals, however, there is a need to explore this vast reserve further for identification of various novel phytochemicals and chemotherapeutic agents for better management of this disease [19,20,21,22,23,24,25,26,27,28,29,30,31,32]. These natural products display inherent anti-cancer properties which emanate from a range of phytochemicals such as alkaloids, diterpenoids, flavonoids, polyphenolic compounds and sesquiterpenes obtained from various medicinal plants, fruits and vegetables [23,27,33,34,35,36,37]. Besides, these herbal medicines sensitize cancers to conventional therapeutic agents by regulating various oncogenic targets such as growth factors, chemokines, inflammatory enzymes and transcription factors; averting the adverse side effects of chemotherapeutic drugs, extending survival time and boosting the quality of life in cancer patients [24,38,39,40].

*Magnolia officinalis*, *Magnolia obovata* and *Magnolia grandiflora* are important traditional Chinese and Japanese herbal plants which possess immense medicinal properties. Magnolia bark has been extensively used as Chinese folklore medicine and is still in use in modern clinical practices [41,42,43,44,45]. Magnolia trees have striking features like their alluring flowers with fragrance, and petiolate leaves containing large stipules surround the stem and later fall, leaving a distinctive scar around the node; the wood of the tree is tough, light weight and easy to work, and is sought after by craftsmen [46]. Historically, the tree was used commonly for gastrointestinal disorders, anxiety, cough, acute pain, and allergic diseases. Magnolol (MAG) is hydroxylated biphenyl isolated from the root and stem bark of Magnolia tree*.* MAG exhibits a huge range of biological activities such as muscle relaxant, anti-oxidative, anti-atherosclerosis, anti-inflammatory, and anti-microbial effects [47,48,49].

Numerous preclinical studies have established that MAG exerts its effect on different types of human cancers such as those of lung, prostate, breast, gall bladder, colon, skin and hepatocellular carcinoma [50,51,52,53,54,55,56,57]. The plausible molecular mechanisms liable for the anti-cancer potential of MAG are reduced cell proliferation or cell cytotoxicity, induction of apoptosis, accumulation of reactive oxygen species (ROS), induction of autophagy and activation/inactivation of various cellular signaling pathways [46]. Several in vitro studies have led to a handful of in vivo studies on different adult animal species which demonstrated that MAG has a good safety profile, reduced tumor growth, induced apoptosis and inhibited invasion, migration and metastasis [56,58,59,60,61]. This review summarizes the underlying molecular mechanisms responsible for the anti-cancer activity that unravels the prospective of MAG as a potent candidate that can be designed and developed into an accomplished anti-cancer drug.

## 2. Chemistry of Magnolol

**Figure 1:**
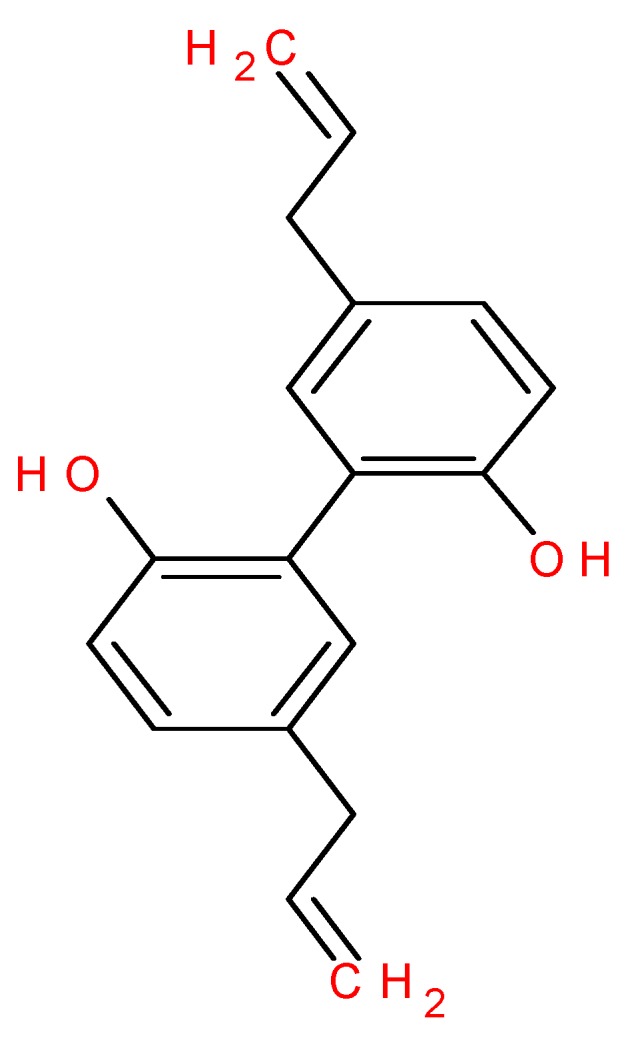
Structure of magnolol.

MAG is a lignan, an organic compound found in the bark of *M. officinalis* or in *M. grandiflora* with a molecular weight of 266.34 g/mol and monoisotropic mass of 266.131 g/mol. The molecular formula of MAG is C_18_H_18_O_2_. The melting temperature of MAG is 101.5–102 degrees Celsius and it is soluble in water at 1.24 mg/L at 25 degrees Celsius. The spectral property shows that the maximum absorption wavelength is at 293 nm [51,62,63,64]. The IUPAC name of MAG is 2-(2-hydroxy-5-prop-2-enylphenyl)-4-prop-2-enylphenol and it is also commonly known as 5,5′-Diallyl-[1,1′-biphenyl]-2,2′-diol; 5,5′-Diallyl-2,2′-biphenyldiol; 5,5′-Diallyl-2,2′-dihydroxybiphenyl; 2,2′-Bichavicol [65]. The structure of MAG is shown in Figure 1. The content of MAG in extracts of magnolia tree is influenced by various environmental factors such as area of origin, altitude of the cultivar, the age of the tree and the part of the plant from where it is extracted [46,66,67,68]. The highest content of MAG was seen in the roots of the tree at a concentration of 87–96 mg/g of extract [66,68]. In view of all the influencing factors, the concentration of MAG varies from 0.05 mg/g to 91.91 mg/g in plant extracts [68]. Various methods can be used for the extraction of MAG from the extract obtained from bark, roots and leaves. These are generally aqueous and/or organic extractions, affecting the retrieval of MAG. Therefore, supercritical extraction, maceration and sonication can be employed to optimize the extraction [69].

## 3. Biological Activities of Magnolol

Several pharmacological active compounds such as magnolol, honokiol, 4-*O*-methylhonokiol, obovatol and few other neolignan compounds are found in the bark of Magnolia tree. MAG is reported to possess an array of pharmacological effects including anti-oxidant [70], anti-inflammatory [71], anti-bacterial [10], anti-thrombotic or anti-platelet [72], anti-stress [73], anti-anxiety, anti-Alzheimer [74], anti-stroke [75], hypoglycemic [76], smooth muscle relaxant [77,78], weight control [79], anti-dyspeptic/prokinetic [80], anti-epileptic [81], and hepatoprotective effects [82]. Small-scale clinical studies on MAG and its interaction with gamma-aminobutyric acid-A (GABA-A) and muscarinic receptors show that it helps in decreasing the anxiety levels in patients [78,83,84,85]. The anti-depressant activity of MAG observed in preclinical studies is due to the alterations in serotonin turnover in the frontal cortex, nucleus accumbens and striatum [86].

MAG can easily cross the blood brain barrier [87,88] and its oral bioavailability is in the region of 10%. MAG is mainly metabolized in the liver with glucuronides as its chief metabolite. Furthermore, acute or long term, preclinical or clinical studies on intake of Magnolia-based preparations did not display any biological alterations. However, very high dosage of MAG may induce hepatotoxicity in vitro [89,90]. Therefore, MAG can be used as a new generation of anti-craving, anti-abstinence, and neuroprotective drugs, with their GABA-ergic activity as well as for the treatment of spasms, convulsions and its associated pain [91]. In the cardiovascular system, it displayed vascular relaxation, anti-atherosclerosis and anti-platelet effects. In the gastrointestinal system, it demonstrated anti-gastric ulcer, anti-esophageal obstruction, hepatoprotective and anti-diarrhea effects [92].

## 4. Molecular Targets of Magnolol

**Figure 2:**
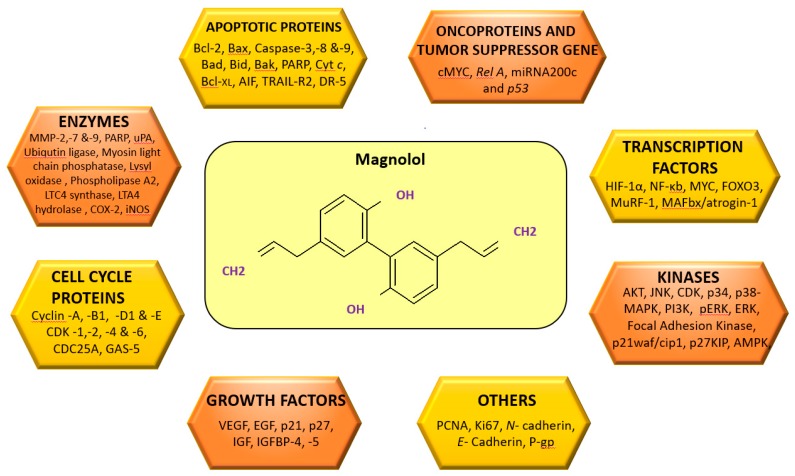
Various molecular targets modulated upon magnolol treatment.

MAG possesses an array of molecular targets that modulate the expression of different genes involved in cancer cell survival, proliferation, invasion, metastasis, chemoresistance and cell death (Figure 2). It is a well-established fact that inhibition of apoptosis is an important strategy for cancer development [37,93,94,95,96]. Release of mitochondrial cytochrome *c* (cyt-*c*) to the cytosol is controlled by a pro-apoptotic B-cell lymphoma protein-2 (Bcl-2) family of proteins such as Bcl-2-associated X protein (Bax), BH3 interacting-domain death agonist (Bid) and Bcl-2 homologous antagonist/killer (Bak) and by the anti-apoptotic Bcl-2 family of proteins such as Bcl-2 and B-cell lymphoma-extra large (Bcl-x_L_) which in turn activate the intrinsic apoptosis pathway. Furthermore, it is also known that activation of caspases play a vital role in apoptosis-mediated cancer cell death [97]. The anti-cancer activity of MAG is linked with the regulation of the caspase cascades and cleaved poly (adenosine diphosphate-ribose) polymerase (PARP) [47,98,99,100,101,102,103]. Yang et al., in the year 2003, reported that MAG increased the expression of Bad, Bcl-_XS_, caspases-3, -6, and -9 and c-Jun N-terminal kinases (JNK) and suppressed the expression of Bcl-x_L_ and extracellular phosphorylated signal-regulated kinase (ERK) in human lung squamous carcinoma [98]. MAG induced apoptosis via the cyt-*c*/caspase-3/PARP/Apoptosis inducing factor (AIF) & phosphatase and tensin homolog (PTEN)/AKT/caspase-9/PARP pathways in CGTH W-2 thyroid carcinoma cell [101]. Furthermore, MAG also induced apoptosis by enhancing the expression of PTEN and down-regulation of AKT [101,104].

MAG also exerts it anti-cancer activity by modulating various proteins involved in the cell cycle regulation [46]. Chen et al., reported that treatment of U373 glioblastoma cells with MAG induced cell cycle arrest at the G0/G1 phase by downregulating the expression of cyclin-A and -D1, and escalating the protein levels of p21/Cip1 [105]. Additionally, treatment of COLO-205 cells with MAG ameliorates the protein expression of p21 thereby inducing cell cycle arrest by inhibiting the cyclin–cyclin dependent kinases (CDKs) system [59].

Constitutive activation of nuclear factor kappa B (NF-κB) down-regulates apoptotic gene and/or upregulates anti-apoptotic gene expression. Furthermore, it also increases the expression of the genes involved in malignant conversion and tumor promotion [8,63,106,107,108,109,110,111,112,113,114,115]. It is now well known that the primary targets of MAG are NF-κB and NF-κB regulated proteins and that MAG induces cell death and reduces cell proliferation by inhibition of NF-κB activity [116,117,118]. MAG prevents invasion and migration of cancer cells by reversal of epithelial-mesenchymal transition (EMT) via inhibition of NF-κB activation. MAG inhibits cancer metastasis by reducing the expression of matrix metalloproteinase-7, -9 (MMP-7, -9) and urokinase plasminogen activator (uPA) [116,119].

MAG activates autophagic cell death by suppressing the levels of phosphorylated AKT and mammalian target of rapamycin (mTOR) [52]. Furthermore, it causes lung cancer autophagy by blocking the Phosphatidylinositol-4,5-bisphosphate 3-kinase (PI3K)/PTEN/AKT pathway [120]. An MAG derivate, Ery5 inhibited angiogenesis and induced cell death via autophagy and not apoptosis in human umbilical cord vein endothelial cells (HUVEC) and PC-3 cells. In addition, treatment with MAG and knocking down of vital autophagic protein ATG7 reversed the Ery5-mediated autophagy and inhibition of angiogenesis [121]. Regulation of all these molecular targets by MAG in different malignancies will be discussed in the next section of this review.

## 5. Cancer Chemopreventive and Therapeutic Properties of Magnolol

**Figure 3:**
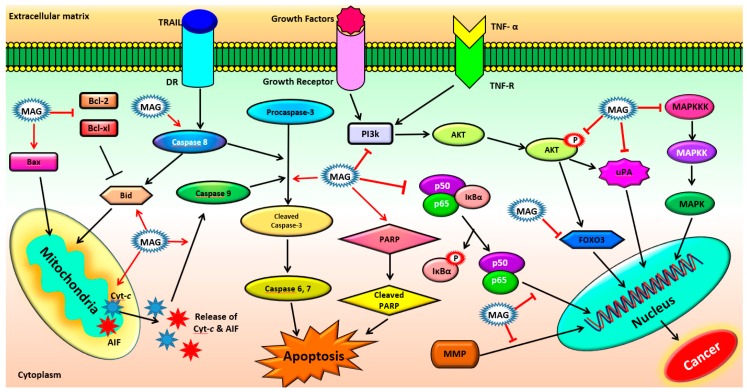
Effect of magnolol on different molecular signaling pathways. (MAG: Magnolol; Τ: Inhibition/Downregulation; **↑**: Activation/Upregulation; Τ: Inhibition/Downregulation by MAG; **↑**: Activation/Upregulation by MAG).

Increasing lines of evidence confirm that MAG controls survival, proliferation, invasion, angiogenesis, metastasis, and chemoresistance of various types of cancers such as bladder cancer, brain cancer, breast cancer, colon cancer, leukemia, liver cancer, lung cancer, ovarian cancer, prostate cancer and skin cancer by regulating multiple signaling pathways (Figure 3). These studies provide a considerable amount of proof that MAG has significant potential as an effective multi-targeted agent for both the prevention and treatment of several cancers and are briefly summarized below.

## 6. Effect of Magnolol in Different Cancers

### 6.1. Bladder Cancer

Approximately 429,800 new cases and 165,100 deaths occurred globally due to bladder cancer in 2012 [1]. Various studies have shown the efficacy of MAG against this cancer (Table 1). Treatment of MAG with the human urinary bladder cancer 5637 cells showed that it promoted apoptosis and arrested the cells at the G2/M phase of the cell cycle. This anti-cancer activity is achieved through downregulation of cyclin and CDK expression and upregulated expression of the CDK inhibitor p27Kip1 [122]. Another study conducted by the same group of scientists revealed that MAG treatment of 5637 bladder cancer cells inhibits expression of MMP-9 induced by Tumor necrosis factor–alpha (TNF-α) by decreasing the binding affinity of the transcription factor NF-κB to the MMP-9 promoter [103]. MAG attenuated angiogenesis in vitro and in vivo which is mediated by inhibition of the expression of hypoxia-inducible factors-1α (HIF-1α) and vascular endothelial growth factor (VEGF) secretion in human bladder cancer cells [123]. In an animal study on bladder cancer-bearing mice, MAG downregulated the expression of transcriptional factor Forkhead box O3 (FoxO3), ubiquitin ligase, MuRF-1 and MAFbx/atrogin-1. MAG has an anti-atrophic effect on cells undergoing chemotherapy [53].

### 6.2. Brain Cancer

Glioblastoma multiforme (GBM) is the most encroaching primary malignant tumor of the central nervous system [158]. A study conducted by Chen L.C. et al., on the effect of MAG has shown it to induce anti-proliferative activity against the U373 human glioblastoma cell line. MAG downregulated the expression of cyclins A and D1 and upregulated the expression of p21/Cip1 which ultimately resulted in cell cycle arrest at the G0/G1 phase [105]. Another group of scientists showed that MAG at a higher concentration of 100 µM induced apoptosis and DNA fragmentation through upregulation of p27Kip1 protein expression in U373 cells both in vitro and in vivo [133]. Preclinical studies on the effect of combination of MAG and honokiol in U87MG and LN229 glioma cells and the human GBM orthotopic xenograft model showed that MAG acts synergistically with honokiol and halts tumor progression by regulating cyclin-A, -D1 and CDK-2, -4, -6 and through induction of autophagy and apoptosis [136]. Furthermore, another in vitro study on LN229 and U87MG glioma cell lines revealed that MAG downregulates myosin light chain phosphatase and *N*-cadherin protein expression level, which plays a pivotal role in cell migration and malignancy [137]. Preclinical studies on treatment of MAG with rat cortical neurons and human neuroblastoma SH-SY5Y cells showed an increase in calcium level in cells via the phospholipase C (PLC)-mediated pathway where calcium is released into the cytoplasm from intracellular storage (Table 1) [60].

### 6.3. Breast Cancer

Breast cancer is the most commonly diagnosed cancer and is one of the leading causes of cancer death in women worldwide [1]. In vitro and in vivo studies on the effect of MAG against cells of the highly invasive human breast cancer cell line MDA-MB-231 and female nude immunodeficient mice revealed that MAG downregulates MMP-9 expression by inhibiting the binding of NF-κB to the MMP-9 promoter [116]. MAG causes cell cycle arrest at the G2/M phase in MCF-7 cells and induces the caspase independent intrinsic apoptotic pathway mediated by enhanced reactive oxygen species (ROS) production, upregulation of proapoptotic proteins like Bax, p21 and p53, down-regulation of anti-apoptotic proteins like Bcl-2, cyclin-B1 and CDK-1 and translocation of cyt-*c* and release of AIF from mitochondria to the cytosol [126]. Hou X. et al., disclosed the anti-proliferative activity of MAG by analytical techniques such as 2D LC-MS, where it was found that MAG inhibits the growth of the MDA-MB-231 cell line [125]. MAG can potentially diminish metastasis by inhibiting enzyme Lysyl oxidase (LOX) and downregulation of focal adhesion kinase expression which is considered as a strong mechanism by which extracellular matrix remodulation takes place during metastasis [124]. Hagiwara K. et al., identified that MAG treatment has the ability to induce novel tumor suppressor microRNA-200c (miRNA-200c) which led to ZEB1 inhibition and *E*-cadherin induction in breast cancer cells (Table 1) [54].

### 6.4. Colorectal Cancer

According to the global cancer statistics 2012, colorectal cancer is the third most common cancer [1]. Interestingly, MAG treatment with colon cancer induced apoptosis by upregulating the expression of the p27Cip1 protein [133]. Park J.B. et al., reported that HCT-116 colon cancer cells upon treatment with MAG activated AMP-activated protein kinase (AMPK), enhanced the expression of pro-apoptotic protein Bax and p53 and downregulated the anti-apoptotic protein Bcl-2 [132]. Another study conducted by Kang Y.J. et al., in 2012 demonstrated that MAG potentially inhibited Wnt3a-mediated β-catenin translocation into the nucleus and suppressed the expression of *c*-myc, MMP-7, and uPA in SW480 and HCT116 human colon cancer cells [119]. In vitro and in vivo studies showed treatment with MAG induced cell cycle arrest at the G1/G0 phase of the cell cycle by increasing the p21 level and decreasing DNA synthesis [131]. Two different studies conducted by the same group indicated that MAG induced apoptosis in COLO205 cells by downregulating the expression of Bcl-2 protein and increasing the cytosolic free Ca (2+) level, cyt-*c* translocation from mitochondria to cytosol and activation of caspase-3, -8 and -9 [57]. It suppressed proliferation of cells by inhibiting DNA synthesis and arrested the cells at the G0/G1 phase of the cell cycle. Furthermore, COLO-205 cells implanted subcutaneously in nude mice upon treatment with MAG led to profound regression of these tumors which was mediated by the increase in the p21 protein expression level and the induction of apoptosis (Table 1) [59].

### 6.5. Leukemia

Leukemia occurs in the tissue that forms blood. The incidence and the mortality rate of this cancer is increasing significantly every year. MAG treatment effectively inhibited proliferation of human HL-60 cells and Jurkat-T leukemia cells by promoting apoptosis in a dose- and time-dependent manner which was mediated through increased cytosolic cyt-*c* concentration, proteolytic cleavage of PARP and activated caspase-2, -3 and -9 activities [139]. Ikai T. et al., in the year 2006 reported that MAG treatment with human leukemia U937 cells induced caspase independent apoptosis by diminishing the mitochondrial membrane potential, Bcl-2 protein expression and ERK signaling pathway [140]. In addition, it also increased the translocation of apoptosis inducing factor (AIF) from mitochondria to the cytosol [140]. MAG was found to exert its anti-cancer activities against human myeloid leukemia HL-60 cells by augmenting the level of Bax and cleavage of caspase-3 and repressing the PI3K/AKT pathway which led to the induction of apoptosis and autophagy [121]. In an in vivo study, treatment of rat basophilic leukemia (RBL)-2H3 cells with MAG showed decreased leukotriene (LT) C4 and LTB4 production. Moreover, MAG also decreased the Ca (2+) level within the cells, resulting in inhibition of two Ca (2+) dependent enzymes, i.e., cytosolic phospholipase A2 (PLA2) and 5-lipoxygenase (5-LO). It also inhibited the functioning of two other enzymes, namely, LTC4 synthase and LTA4 hydrolase which are essential for LT-synthesis (Table 1) [138].

### 6.6. Liver Cancer

Liver cancer accounts for second highest death from cancer globally [1,159]. Many in vitro *and* in vivo investigations offer evidence of the effectiveness of MAG against liver cancer where it is found to increase cell cytotoxicity, repress cell proliferation/cell viability and reduce tumor growth significantly [61,127,129,141,142,143]. MAG induced apoptosis in HepG2 cells by increasing the intracellular level of calcium along with increased translocation of cyt-*c* from mitochondria to the cytosol and activation of caspase-3, -8, and -9 [57]. Another in vitro study on the same cell line conducted by the same group displayed enhanced apoptosis by upregulation of the p21 protein and inhibition of DNA synthesis. Therefore, it arrested the cell cycle progression at the G0/G1 phase of the cell cycle [59]. Furthermore, Maioli M. et al., in 2018, reported that modifying the MAG hydroxyl group into a suitable ester derivative showed a decrease in hepatic tumor malignancy (Table 1) [51].

### 6.7. Lung Cancer

Lung cancer is the leading cause of death in males and has surpassed breast cancer as the leading cause of cancer death among females [1]. MAG is known to repress cell proliferation and reduce tumor growth, invasion and metastasis in lung cancer (Table 1) [61,144]. Non-small cell lung cancer cell lines (NSCLC) such as A549, H441 and H520 upon treatment with MAG increased DNA fragmentation, exhibited a change in mitochondrial membrane potential and release of pro-apoptotic proteins like Bid, Bax and cyt-*c* from mitochondria resulting in the induction of apoptosis. Further, it also helped in the nuclear translocation of AIF, activation of endonuclease G and cleavage of PARP (caspase independent apoptotic pathway) [146]. In vitro studies on A549 and H1299 cells showed that MAG causes cell cycle arrest at the G0/G1 phase while simultaneously upregulating pro-apoptotic proteins expression, including TRAIL-R2 (DR5), Bax, caspase-3, cleaved caspase-3, and cleaved PARP. Further, in the same study, the scientists reported that in vivo A549 xenograft model upon treatment with MAG suppressed tumor growth and induced apoptosis by epigenetically activating DR5, which in turn activated death receptor-mediated apoptosis [145]. Seo J.U. et al., in 2011, revealed that MAG can alter the cell cycle in A549 cells and can also mediate caspase-dependent apoptosis via downregulation of NF-κB/Rel A in the nucleus [118]. Another study on small lung cancer H460 cells demonstrated that MAG initiates cell death via autophagy instead of apoptosis [120]. Ahn K.S. et al., reported that MAG inhibited NF-κB activation in H1299 cells [117]. MAG treatment inhibited proliferation and induced apoptosis of CH27 cells through downregulation of the Bcl-2 family, increase in cytosolic cyt-*c* and activation of caspase-9, -3 and -6 [98]. In vitro studies on A549 cells confirmed that MAG causes cell cycle arrest at the mitotic phase by inhibiting microtubule polymerization, and in vivo studies on the xenograft model of human A549 NSCLC tumor showed a reduction in tumor growth and size [52].

### 6.8. Ovarian Cancer

Although the rate of incidence of ovarian cancer is not as high as breast cancer and lung cancer, it remains one of the leading causes of deaths due to cancer among women. MAG effectively induced cell cytotoxicity and reduced cell proliferative activity in OVCAR-3 cells [129]. MAG treated with HER2-overexpressing ovarian cancer cells showed downregulation of HER2 mRNA expression mediated by the suppression of VEGF, MMP-2, cyclin-D1 proteins and the PI3K/AKT/mTOR-signaling pathway and enhancement in PARP cleavage and activated caspase-3 [149]. It was evident from the report of Han H.K. et al., that MAG significantly reduced multidrug resistance (MDR) via the downregulation of phosphorylated-glycoprotein (P-gp) expression (Table 1) [150].

### 6.9. Prostate Cancer

Approximately 1.1 million new cases of prostate cancer occurred in 2012, and this is the second most frequently diagnosed cancer in men worldwide [1,160]. Several preclinical studies have shown the efficacy of MAG against prostate cancer. MAG treatment of PC-3 cells can potentially induce apoptosis by decreasing the concentration of phosphorylated AKT and the epidermal growth factor receptor (EGFR) signal transduction pathway. Further, it decreased phosphorylation of serine 136 of Bad protein, assisted in the translocation of Bax to mitochondria and promoted the release of cyt-*c*, which in turn activated downstream caspase cascade to induce apoptosis [152]. MAG diminishes cell proliferation activity by autophagy and inhibits angiogenesis in PC3 cells [121]. Hwang E.S. et al. reported that MAG suppressed the metastatic property of PC-3 cells via downregulation of MMP-2, -9 both at the transcriptional and translational levels [153]. In vitro studies on androgen insensitive prostate cancer cell lines DU 145 and PC3 cells disclosed that MAG treatment causes cytotoxicity and affects the cell cycle progression by arresting the cells at the G2/M phase of the cell cycle by suppressing the expression of cell cycle regulatory proteins such as cyclin-A, -B1, -D1 and -E, and kinases like CDK-2 and CDK-4 [55]. The same research team performed another preclinical study on LNCap and PC3 cells and revealed that treatment with MAG downregulated the expression of Insulin-like growth factor-1 (IGF-1) and associated proteins such as insulin-like growth factor binding Protein-5 (IGFBP-5) and IGFBP-4 (Table 1) [151].

### 6.10. Skin Cancer

Malignant melanoma of the skin is an important global health problem. It is the most commonly diagnosed cancer, found predominantly in the white population [161]. Various preclinical studies showed MAG to be effective against skin cancer. A study conducted by Wang T.H. et al., reported that MAG induced apoptosis by upregulating the expression of the long non-coding RNA of growth arrest-specific 5 (GAS5) [154]. Further, MAG treatment can prevent chemically and UVB-induced skin cancer by inducing apoptosis [157]. MAG inhibits the expression of inducible nitric oxide synthase (iNOS), cyclooxygenase-2 (COX-2) and nuclear translocation of the NF-κB subunit thereby reducing its efficacy to bind with DNA. Furthermore, MAG also suppressed ERK1/2 kinase, MAPK, and the PI3K/AKT pathway in DMBA/TPA-induced skin cancer in female mice [155]. MAG inhibited cell proliferation in the human malignant melanoma A375-S2 cell line by increasing caspases-3, -8,-9 activities, augmenting the expression of anti-apoptotic mitochondrial protein Bcl-2 while decreasing the expression of pro-apoptotic protein Bax [147]. In vivo studies on different animal models of skin cancer demonstrated that MAG reduced tumor growth, induced apoptosis and arrested cell cycle at the G2/M phase (Table 1) [56,156,157].

### 6.11. Other Cancers

As discussed above, MAG possesses a potent anti-cancer effect against different types of cancers. In addition to the above-mentioned cancers, it has been found to be effective against other cancers as well such as gall-bladder cancer, fibrosarcoma, oral cancer, thyroid cancer, cholangiocarcinoma, cervical cancer, gastric cancer, kidney cancer and spleen cancer (Table 1). However, only a handful of literature is available on the effect of MAG in these cancers. Gallbladder cancer is a relatively rare cancer and the prevalence of this cancer shows geographical and racial variations. It is common in central and eastern Europe, central and South America, Japan and northern India [162]. MAG downregulated the expression of cyclin-D1, CDC25A, and CDK-2 protein and upregulated the expression of p53 and p21 proteins in human gallbladder cancer cell lines GBC-SD and SGC-996. Further, the in vivo study showed that MAG treatment of BALB/c homozygous nude mice reduced tumor growth significantly [58].

Fibrosarcoma, commonly known as fibroblastic sarcoma, is a malignant mesenchymal tumor which originates from fibrous connective tissue. MAG efficiently reduced malignancy in human fibrosarcoma cell line HT-1080 through inhibition of MMP-9 activity [134]. In 2012, approximately 300,400 new cases and 145,400 deaths occurred due to oral cancer globally [1]. An investigation on the efficacy of MAG against OC2 oral cancer cells showed that it increases Ca (2+) concentration within the cells via PLC dependent endoplasmic reticulum release and Ca (2+) influx via store-operated Ca (2+) channels (SOC) activated by protein kinase C (PKC) [148]. Thyroid cancer is a cancer that initiates from the tissues of the thyroid gland and gradually the rate of cancer incidence is increasing every year. It was reported by Huang et.al that MAG treatment of CGTH W-2 thyroid carcinoma cells, robustly induced apoptosis by augmenting the expression of activated caspases. Apoptosis was mediated by the cyt-*c*/caspase-3/PARP/AIF and PTEN/AKT/caspase-9/PARP pathways whereas necrosis induced by MAG occurred via PARP activation [101]. Gastric cancer is the fourth most commonly diagnosed cancer in the world. The effects of MAG on SGC-7901 gastric cancer cells showed that it induced morphological changes in the cells and its cytotoxic effects were associated with DNA damage, the mitochondrial-mediated apoptosis pathway, increased ratio of Bax/Bcl-2, dissipation of mitochondrial membrane potential and sequential activation of caspase-3 and inhibition of PI3K/AKT-dependent pathways [135].

Cholangiocarcinoma is a malignancy that arises primarily from the epithelial lining of the bile duct. Treatment of cholangiocarcinoma CCA cells with MAG decreased malignancy and proliferation of the cells by downregulation of PCNA, Ki67, MMP-2, -7 and -9 protein expression and inhibition of the NF-κB signaling pathway [130]. Around 265,700 deaths occurred worldwide due to cervical cancer in 2012. It is the third leading cause of cancer death among females in less developed countries [163]. Two different studies conducted by Li M. et al., and Syu W.J. et al., on Hela cells reported that MAG increased cell cytotoxicity and reduced the cell survival capability of the cancer cells [127,129]. Moreover, MAG strongly inhibited TNF-α stimulated NF-κB activation and prevented MDR in KB/MDR1 cells by decreasing P-gp expression [128]. Kidney cancer, generally known as renal cancer, is a type of cancer that originates in the cells of the kidney [164]. MAG displays potent anti-cancer activity against human renal tubular ACHN cells [127]. Spleen cancer is a very rarely occurring cancer that develops in the spleen. Ikeda K. et al., in 2003, suggested that treatment with MAG in vivo displayed a substantial reduction in tumor growth, invasion and metastasis [61].

## 7. Conclusions

MAG, honokiol, 4-*O*-methylhonokiol, obovatol and other neolignans found in the bark of Magnolia tree are some of the principle compounds that confer medicinal qualities to the plant. MAG, an organic compound (lignan) isolated from various Magnolia species, has been studied extensively for its biological activities such as anti-oxidant, anti-inflammatory, anti-bacterial, anti-thrombotic or anti-platelet, anti-stress, anti-anxiety, anti-Alzheimer, anti-stroke, hypoglycemic, smooth muscle relaxant, weight control, anti-dyspeptic/prokinetic, anti-epileptic and hepatoprotective activities. Numerous preclinical studies on MAG have shown its cytotoxic potential against different cancers and other medical conditions. Through several molecular mechanisms, MAG suppressed the pathogenesis and repressed the spread of cancer in vitro and in vivo. It acts via onset of the tumor suppressor p53 pathway and inhibition/downregulation of tumor progression NF-κB, Wnt/β-catenin, PI3K-AKT and MAPK/ERK pathways.

The molecular targets associated with MAG activity are enzymes, apoptotic proteins, transcription factors, growth factors, oncoproteins, tumor suppressor genes, receptors, and other proteins involved in cell proliferation, cellular differentiation, survival, angiogenesis, migration, and invasion, or other cellular processes involved in cancer. Various animal studies strongly advocate the potential role of MAG in controlling the growth of different tumors. However, not even one clinical study has investigated the efficacy of MAG. As MAG is obtained from Mother Nature, it could drastically economize the expenditure associated with this ever-growing dreadful disease. However, additional preclinical and clinical investigations are essential to proclaim the therapeutic potential of MAG that would help to bring this compound to the clinic.

## Figures and Tables

**Table 1:** Magnolol (MAG) and its mechanism of actions against different cancers.

Cancer	Models	Mechanism(s) of Action	References
Bladder cancer	In vivo	↓Myostatin, activin A formation, FoxO3, ubiquitin ligases MuRF-1 & MAFbx/atrogin-1	[53]
	In vitro	↑p27Kip1 ↓cyclin -B1/CDC2	[122]
	In vitro	↓MMP-9	[103]
	In vitro	↓HIF-1α/VEGF-dependent angiogenesis pathways	[123]
	In vivo	↓HIF-1α/VEGF-dependent angiogenesis pathways	[123]
Breast cancer	In vitro	↑miR-200c & *E*-cadherin	[54]
	In vitro	↓LOX	[124]
	In vitro	↓Cell growth	[125]
	In vitro	↑Cell cycle arrest at G2/M phase, ROS, release of cyt-*c*, AIF, Bax, p21 & p53 ↓MMP, Bcl-2, cyclin-B1 & CDK-1	[126]
	In vitro	↓MMP-9 & NF-κB activity	[116]
	In vivo	↓MMP-9 & NF-κB activity	[116]
Cervical cancer	In vitro	↓Cell survival	[127]
	In vitro	↓P-gp & MDR	[128]
	In vitro	↑Cell cytotoxicity	[129]
Cholangiocarcinoma	In vitro	↓PCNA, Ki67, MMP-2,-7,-9, cyclin-D1, p-IκBα & p-P65 ↑Cell cycle arrest in G1 phase	[130]
	In vivo	↓Tumor growth	[130]
Colon cancer	In vitro	↑Cytosolic free Ca(2+); translocation of cyt-*c*; caspase-3, -8, & - 9 ↓Bcl-2	[57]
	In vitro	↓DNA synthesis ↑cell cycle arrest at G0/G1 phase	[59]
	In vivo	↓Tumor growth ↑p21	[59]
	In vivo	↑ERK phosphorylation, p21 ↓thymidine incorporation	[131]
	In vitro	↓β-catenin, MMP-7, uPA & *c*-myc	[109]
	In vivo	↓Invasion & motility of tumor cells	[109]
	In vitro	↑p53, Bax & AMPK activation ↓Bcl-2	[132]
	In vitro	↑Apoptosis & p27Cip1 protein	[133]
Fibrosarcoma	In vitro	↓MMP-9	[134]
Gallbladder cancer	In vitro	↑Cell cycle arrest at G0 /G1 phase, p53 & p21 ↓cyclin -D1, CDC25A, & CDK-2	[58]
	In vivo	↓Tumor growth ↑cell cycle arrest at G0 /G1 phase, p53 & p21 ↓cyclin -D1, CDC25A & CDK-2	[58]
Gastric cancer	In vitro	↓PI3K/AKTsignaling pathways	[135]
Glioblastoma	In vitro	↓Cyclin-A, -D1 & CDK-2, -4& -6	[136]
	In vitro	↓Tumor growth ↑apoptosis	[136]
	In vitro	↑Cell cycle arrest at G0 /G1 phase& p21/Cip1 ↓cyclins -A & -D1& DNA synthesis	[105]
	In vitro	↑p27Kip1 & apoptosis	[133]
	In vivo	↑p27Kip1 & apoptosis	[133]
	In vitro	↓myosin light chain phosphatase & *N*-cadherin	[137]
Kidney cancer	In vitro	↓Cell survival	[127]
	In vivo	↓Tumor growth, invasion & metastasis	[61]
Leukemia	In vivo	↓LTs, PLA2, 5-LO, LTC4 synthase & LTA4 hydrolase	[138]
	In vitro	↑Bax & cleavage of caspase-3, ↓PI3K/AKT pathway	[121]
	In vitro	↑Apoptosis, cyt-*c* release, caspase-9,-3 &-2 & cleaved PARP	[139]
	In vitro	↓ERK signal transduction &Bcl-2 protein ↑AIF	[140]
Liver cancer	In vitro	↓Cell viability	[51]
	In vitro	↓Cell survival	[127]
	In vitro	↓Cell proliferation	[141]
	In vitro	↓Cell viability	[142]
	In vitro	↑Cytosolic free Ca (2+), translocation of cyt-*c*, caspase-3, -8, & -9 ↓Bcl-2	[57]
	In vitro	↓DNA synthesis ↑cell cycle arrest at G0/G1 phase& apoptosis	[59]
	In vivo	↓Tumor growth, invasion & metastasis	[61]
	In vitro	↑Cell cytotoxicity	[129]
	In vitro	↑Cell cytotoxicity	[143]
Lung cancer	In vitro	↑Cell cycle arrest in M phase, polymerization of microtubule, apoptosis via p53-independent pathway & autophgy via ↓AKT/mTOR	[52]
	In vivo	↓Tumor growth	[52]
	In vitro	↓Cell proliferation	[144]
	In vitro	↑Cell apoptosis cell cycle arrest in G0/G1 phase, TRAIL-R2 (DR5), Bax, caspase-3, & cleaved PARP	[145]
	In vivo	↓Tumor growth	[145]
	In vitro	↑Bad, Bcl-_XS_, & caspase-9, -3 & -6↓Bcl-x_L_	[98]
	In vivo	↓Tumor growth, invasion & metastasis	[61]
	In vitro	↓NF-κB activation	[117]
	In vitro	↑Autophagy ↓PI3K/PTEN/AKT pathway	[120]
	In vitro	↑Caspase-3 & cleavage of PARP↓NF-κB/Rel A	[118]
	In vitro	↑Release of Bid, Bax & cyt-*c* from mitochondria ↑PI3K/AKT & ERK1/2	[146]
Melanoma	In vitro	↑Casapase-3, -8, -9 activities	[147]
Neuroblastoma	In vivo	↑Cytosolic free Ca (2+); via PLC-mediated pathway	[60]
Oral cancer	In vitro	↑Ca (2+) influx via PKC-sensitive store-operated Ca (2+) entry & ↑Ca (2+) release from ER in a PLC-associated manner	[148]
Ovarian cancer	In vitro	↑Cell cytotoxicity	[129]
	In vitro	↓PI3K/AKT/mTOR-signaling, ↑PARP cleavage, caspase-3 activation	[149]
	In vitro	↓P-gp	[150]
Prostate cancer	In vitro	↓IGF-1, IGFBP-5, p-IGF-1R & ↑IGFBP-3, IGF-1R	[151]
	In vitro	↑Cell cytotoxicity, ↓cyclins -A,- B1,-D1 & -E, ↓CDK-2 & -4	[55]
	In vitro	↓Inhibiting the EGFR/PI3K/AKT signaling, ↑cyt-*c* release, Bax	[152]
	In vitro	↓MMP-2 & MMP-9	[153]
	In vitro	↑Autophagy; ↓cell proliferation, migration, invasion & tube formation	[121]
Skin cancer	In vitro	↑GAS5 & apoptosis	[154]
	In vivo	↓Tumor growth	[56]
	In vivo	↓ERK-1/2; MAPK; PI3K/AKT, iNOS & COX-2	[155]
	In vivo	↑Cleavage of caspase-8 & PARP, p21 & G2/M phase cell cycle arrest	[156]
	In vitro	↑G2/M phase cell cycle arrest, Cip/p21, cleavage of caspase-8 & PARP, ↓cyclin -B1, -A, CDK-4, CDC2	[156]
	In vivo	↓Cell viability & proliferation↑apoptosis	[157]
	In vitro	↓Cell proliferation, Bax & Bcl-2 ↑apoptosis & caspases-3, 8, 9	[147]
Spleen cancer	In vivo	↓Tumor growth, invasion & metastasis	[61]
Thyroid cancer	In vitro	↑Apoptosis via the cyt-*c*/caspase-3/PARP/AIF & PTEN/AKT/caspase-9/PARP pathways & necrosis via PARP activation	[101]

## Abbreviations

AIF	apoptosis inducing factor
AMPK	AMP-activated protein kinase
Bak	Bcl-2 homologous antagonist/killer
Bax	Bcl-2-associated X protein
Bcl-2	B-cell lymphoma 2
Bcl-_XL_	B-cell lymphoma-extra large
Bid	BH3 interacting-domain death agonist
Ca (2+)	Calcium
CDC25A	cell division cycle 25 homolog A
CDK	cyclin-dependent kinase
Cip1	CDK-interacting protein 1
COX-2	Cyclooxygenase-2
cyt-*c*	cytochrome-*c*
DNA	Deoxyribo nucleic acid
DR5	Death receptor 5 EGFR: epidermal growth factor receptor
ERK	extracellular phosphorylated signal-regulated kinase
FoxO3	Forkhead box O3
GAS5	growth arrest-specific 5 HIF-1α:hypoxia-inducible factors-1α
IGF-1	Insulin-like growth factor 1
IGFBP-5	Insulin-like growth factor binding Protein-5
iNOS	inducible nitric oxide synthase
Kip1	Kinase inhibitory protein
5-LO	5-lipoxygenase
LOX	Lysyl oxidase
LT	Leukotriene
MDR	Multidrug resistance
MMP	Matrix metalloproteinases
mTOR	mammalian target of rapamycin
NF-κB	Nuclear factor kappa B
NSCLC	Non-small cell lung cancer cell lines
PARP	Poly ADP ribose polymerase
PCNA	Proliferating cell nuclear antigen
P-gp	Phosphorylated-glycoprotein
PI3K	Phosphatidylinositol-4,5-bisphosphate 3-kinase
PKC	protein kinase C
PLA2	phospholipase A2
PLC	phospholipase C
PTEN	phosphatase and tensin homolog
SOC	Store-operated Ca (2+) channels
TNF-α	Tumor necrosis factor-alpha
TRAIL	TNF-related apoptosis-inducing ligand
uPA	urokinase plasminogen activator
VEGF	Vascular endothelial growth factor
